# Synthesis and Biomedical Applications of Lanthanides-Doped Persistent Luminescence Phosphors With NIR Emissions

**DOI:** 10.3389/fchem.2020.608578

**Published:** 2020-12-14

**Authors:** Xinyuan Qin, Jie Wang, Quan Yuan

**Affiliations:** ^1^Key Laboratory of Biomedical Polymers of Ministry of Education, College of Chemistry and Molecular Sciences, Wuhan University, Wuhan, China; ^2^State Key Laboratory of Chemo/Biosensing and Chemometrics, College of Chemistry and Chemical Engineering, Institute of Chemical Biology and Nanomedicine (ICBN), Hunan University, Changsha, China

**Keywords:** lanthanides, persistent luminescence, near-infrared, bioimaging, therapy

## Abstract

Persistent luminescence phosphors (PLPs) are largely used in biomedical areas owing to their unique advantages in reducing the autofluorescence and light-scattering interference from tissues. Moreover, PLPs with long-lived luminescence in the near-infrared (NIR) region are able to be applied in deep-tissue bioimaging or therapy due to the reduced light absorption of tissues in NIR region. Because of their abundant election levels and energy transfer channels, lanthanides are widely doped in PLPs for the generation of NIR persistent emissions. In addition, the crystal defects introduced by lanthanides-doping can serves as charge traps in PLPs, which contributes to the enhancement of persistent luminescence intensity and the increase of persistent time. In this paper, the research progress in the synthesis and biomedical applications of lanthanides-doped PLPs with NIR emissions are systematically summarized, which can provide instructions for the design and applications of PLPs in the future.

## Introduction

Persistent luminescence phosphors (PLPs) are photoluminescent materials that will remain luminescence after the excitation light is extinguished (Yang et al., 2016; Feng et al., 2018). It is generally accepted that the crystal defects in PLPs can store photogenerated electrons and holes during excitation. After the excitation light is closed, the electrons and holes in the defects can escape from the defects under stimulation, and their recombination generates the persistent luminescence phenomenon (Wang et al., 2018). The delayed luminescence in PLPs enables researchers to completely avoid the interference of autofluorescence in biological samples, which greatly improves the signal-to-noise ratio (SNR) of bioimaging (Rosticher et al., 2016; Gong et al., 2017). In addition, PLPs with near-infrared (NIR) emissions provides the possibility for bioimaging in deep tumor tissues. In 2007, Chermont et al. first applied PLPs to bioimaging, and they realized long-term *in-vivo* imaging for more than 1 h by using the delayed luminescence of PLPs (Chermont et al., 2007). Nowadays, persistent luminescence materials are widely studied in biomedical fields, including biosensing, bioimaging, and tumor therapy.

Generally, PLPs consist of host materials and the doped ions (Hu et al., 2018). The host materials usually display broad emission peaks at 350–600 nm, so their applications in bioimaging and so on are limited. Doping is an effective way to generate narrow band emission at different wavelengths in PLPs (Hai et al., 2020). Lanthanides have a lot of electron energy levels and long-lived excitation states, which can generate a variety of radiation absorption and emission (Zhao L. et al., 2019). Lanthanides-doping is often used to generate the desired visible or NIR emissions in PLPs. Moreover, the doped lanthanides can participate in the charge trapping and detrapping processes. These doped lanthanides may enhance the persistent luminescence intensity and prolong the persistent time (Zhang et al., 2019). In 1996, Matsuzawa et al. reported the milestone SrAl_2_O_4_:Eu^2+^, Dy^3+^ PLPs with bright and durable persistent luminescence (Matsuzawa et al., 1996). In SrAl_2_O_4_:Eu^2+^, Dy^3+^, the Eu^2+^ is the emission center, and Dy^3+^ participates in the charge trapping/detrapping processes. Inspired by Matsuzawa's work, many different lanthanides-doped PLPs have been synthesized, whose applications in biomedical areas have also been investigated (Zhang et al., 2015).

Recently, there are several good reviews on PLPs (Singh, 2014; Liang et al., 2019; Lin et al., 2020). In these reviews, the luminescence mechanisms, the different kinds of PLPs, the controlled synthesis of persistent luminescence materials, and the design of persistent luminescence nanoprobes for biomedical applications have been systematically reviewed (Lin et al., 2019; Ma et al., 2019). Whereas, the design of lanthanides-doped PLPs with NIR emissions and their biomedical applications have not been overviewed. In this review, the development of lanthanides-doped PLPs in recent years is systematically introduced from two aspects: synthesis and biomedical application. This paper mainly reviews the controlled synthesis of lanthanides-doped PLPs with emissions in the NIR region, and the applications of PLPs in biosensing, bioimaging, drug delivery, and phototherapy, which may provide instructions for the future studies on lanthanides-doped PLPs.

## Synthesis of Lanthanides-Doped PLPs With NIR Emissions

Fluorescence imaging in the NIR window (650–1,700 nm) has been widely used in the field of biotechnology, such as bioimaging and targeted disease therapy (Gong et al., 2019). Generally, NIR biological window can be divided into NIR-I (650–1,000 nm) region and NIR-II (1,000–1,700 nm) region according to the wavelength (Liu Y. et al., 2019). Some PLPs whose emission located in the NIR biological window will have deep tissue penetration depth in bioimaging (Zhao H. et al., 2019). Due to having various electron energy levels, lanthanides are highly efficient in producing emissions in the NIR region. This section will introduce the methods for the design and synthesis of lanthanides-doped PLPs with NIR emissions.

### Lanthanides-Doped PLPs With Emissions in NIR-I Region

In the process of NIR luminescence, the doped ions can be used as the luminescence centers to generate corresponding emissions (Zhang et al., 2018). Lanthanides atoms have abundant electron energy levels, long-lived excited states, and more than 200,000 transition channels, which can produce various radiation absorption and emission. Therefore, lanthanides-doping is widely used in the design of NIR luminescence materials, including PLPs. Li et al. prepared lanthanides-doped SrZrO_3_:Yb^3+^ PLPs by high-temperature solid-state reaction (HTSSR) (Li Z. et al., 2018). This phosphor could produce stable luminescence at around 986 nm under UV excitation. Calculations showed that the luminescence at 986 nm was originated from the doped Yb^3+^:^2^F_2/5_-^2^F_2/7_. The NIR persistent luminescence at 986 nm was also detected in SrZrO_3_:Yb^3+^, and the electrons stored in oxygen vacancies was proved to generate the persistent luminescence. Additionally, they showed the persistent luminescence intensity in SrZrO_3_:Yb^3+^ was related to the concentration of the doped Yb^3+^. The NIR persistent luminescence intensity increased with raising the concentration of Yb^3+^, whereas a high concentration of Yb^3+^ resulted in quench of the NIR luminescence. The optimal concentration of the doped Yb^3+^ in SrZrO_3_:Yb^3+^ was determined to be 2.5%. This work showed the good promise of lanthanides in the generation of NIR persistent luminescence by serving as the luminescence centers.

**Figure 1:**
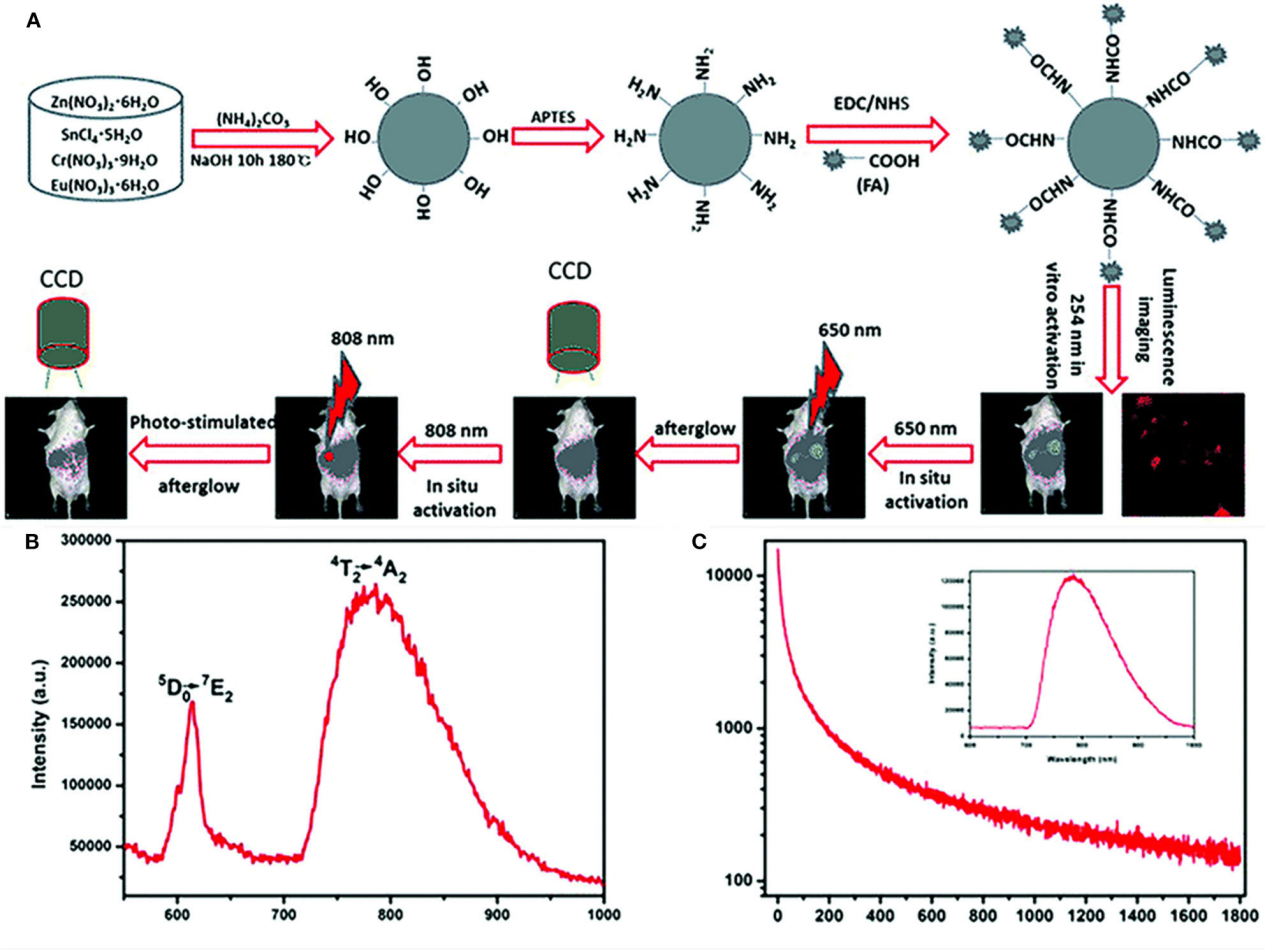
**(A)** Schematic diagram of the synthesis and bioimaging applications of the ZnSn_2_O_4_:Cr,Eu PLPs, **(B)** emission spectrum of the PLPs of excitation at 254 nm, **(C)** decay curve of the PLPs of excitation at 254 nm and the inset is PersL spectrum. Reproduced from a literature Li et al. (2017) with permission from Royal Society of Chemistry.

In addition to being luminescence centers, lanthanides can also serve as defects to regulate the energy storage and transfer process. Li et al. directly synthesized ZnSn_2_O_4_:Cr, Eu PLPs by a hydrothermal reaction (Figure 1A) (Li et al., 2017). In ZnSn_2_O_4_:Cr,Eu, Cr^3+^ acts as the NIR luminescence center with emission at 800 nm (Figure 1B), and Eu^3+^ acts as the trap center to capture photo-generated electrons and generate persistent luminescence after excitation ceases (Figure 1C). The ZnSn_2_O_4_:Cr,Eu PLPs can effectively avoid the light scattering interference and show deep-tissue penetration in bioimaging. Moreover, the authors realized the covalent modification of folic acids on the ZnSn_2_O_4_:Cr,Eu surface for target tumor imaging.

Generally, PLPs need to be charged by UV light, and this largely limits their biomedical applications. Considering the deep penetration of NIR light, the PLPs which are excited by NIR light have a better promise in biomedical areas. Qin et al. successfully synthesized a upconversion PLPs Zn_1.3_Ga_1.4_Sn_0.3_O_4_:Yb^3+^,Er^3+^,Cr^3+^ by HTSSR (Qin et al., 2019). Due to the tailored energy transfer (ET) between doping ions, this phosphor shows the NIR emission of Cr^3+^. In these PLPs, Yb^3+^ first absorbs NIR (980 nm) photons and transfers the energy to Er^3+^ by upconversion (UC) emission, and then Er^3+^ transfers the energy to Cr^3+^ by ET to realize NIR persistent luminescence emission of Cr^3+^ at 694 nm. Due to the special luminescence property, these PLPs will reduce photodamage to biological tissues as well as improving the penetrability of the excitation light, which provides the possibility for deep tumors imaging and therapy.

### Lanthanides-Doped PLPs With Emissions in NIR-II Region

As previously introduced, a longer emission wavelength of PLPs can effectively improve the penetration depth of bioimaging (Zhang et al., 2018). Therefore, the PLPs in NIR-II have a better application prospect in biomedical fields. Xu et al. synthesized the Y_3_Al_2_Ga_3_O_12_:Er^3+^,Cr^3+^ PLPs with emissions in NIR-II region by HTSSR (Xu et al., 2018b). Due to the ET from Cr^3+^ to Er^3+^, these PLPs have the persistent luminescence of both Cr^3+^ (690 nm) and Er^3+^ (1,532 nm). The authors also proved that PLPs did have deep penetration depth in tissues, which improved SNR of bioimaging. In addition, by doping Ho^3+^ in LaAlO_3_ and LaGaO_3_ perovskite, Xu et al. also successfully developed two kinds of persistent luminescence perovskite particles with multi-wavelength emissions (NIR-I and NIR-II) (Xu et al., 2018a). This work indicated that the method of lanthanides doping can be used to adjust the emission wavelength of various types of PLPs. Therefore, this lanthanides co-doping method provides a reference for the synthesis of other PLPs with emissions in NIR-II.

Recently, Xu et al. synthesized a Y_3_Al_2_Ga_3_O_12_:Nd^3+^, Ce^3+^,Cr^3+^ PLPs with multi-wavelength emissions at about 880, 1,064, and 1,335 nm by HTSSR (Xu et al., 2015). Calculations showed that the multi-wavelength luminescence was originated from the doped Nd^3+^:^4^F_3/2_ to ^4^I_9/2_, ^4^I_11/2_, and ^4^I_13/2_, respectively. Since its emissions match with the NIR-I and NIR-II region, PLPs with long persistent luminescence have promising applications in bioimaging and tumor therapy.

In addition to inorganic materials, organic lanthanides co-doped system also shows the persistent luminescence phenomenon. Li et al. synthesized an organic Er^3+^ complex Er(F-TPIP)_3_ [tetrakis(pentafluorophenyl)imidodiphosphinate] successfully (Li H.-F. et al., 2020). This complex had the NIR emission at around 1,500 nm due to the ^4^I_13/2_-^4^I_15/2_ transition of Er^3+^. The lifetime of the Er(F-TPIP)_3_ is 2.65 ms, which was the longest Er^3+^ lifetime in the hydrogenous organic environment. The authors realized the enhancement of Er^3+^ emission through the co-doping of a photosensitizer (phosphororganic molecule), whose enhancement effect was up to 1,600 times. This photosensitizer and lanthanides co-doped method is expected to realize the design and synthesis of organic lanthanides system with strong NIR persistent emissions.

## Biosensing Based on Lanthanides-Doped PLPs

PLPs are ideal materials for building fluorescent probes for biosensing due to their delayed luminescence properties (Kumar et al., 2017; Zhang X. et al., 2018). Li et al. synthesized the PLPs nanoprobe Ir(III)@SiNPs-Eu^3+^ with 653 nm emission for tetracycline (TC) detection (Li X. et al., 2020). Since TC will enhance the emission intensity of doped Er^3+^ and quench the original Ir(III)@SiNPs luminescence, this nanoprobe could realize the ratiometric analysis of the TC in complicated tissues. The authors demonstrated that the nanoprobe was sensitive to TC in the serum background, and the TC-nanoprobe complex could also detect Hg^2+^ sensitively through the ratiometric luminescence mode. In addition, the nanoprobe had low cytotoxicity. The developed nanoprobe has a great application potential in biological detection, including the detection of TC and Hg^2+^ in biological samples.

Recently, Dou et al. synthesized a La_2_O_2_CO_3_:Eu^3+^,Ho^3+^ PLPs with emission at around 704 nm, whose size and persistent luminescence could be adjusted by changing the reaction conditions such as time and reaction power (Dou et al., 2019). They found that the PLPs had good stability in water, which can achieve long-term preservation in liquid environment for more than 1 week. In addition, they demonstrated the La_2_O_2_CO_3_:Eu^3+^,Ho^3+^ nanoprobe could achieve highly sensitive detection of H_2_O_2_ in the serum environment because the H_2_O_2_ can quench the luminescence of this probe. Glucose, on the other hand, can produce H_2_O_2_ through enzymatic (glucose oxidase) reaction *in vivo*, so this probe can determine the serum glucose concentration. Different from traditional detecting methods based on enzymes, this PLP-based method isn't sensitive to the change of temperature and pH. Therefore, this probe will have a broader prospect in the clinical monitoring of patients with hyperglycemia or diabetes.

The application of PLPs has also been extended to the field of food safety. Liu et al. successfully synthesized a kind of persistent luminescence nanophosphors (PLNPs, ZnGa_2_O_4_:Ga,Er,Yb)@MIP (molecularly imprinted) with 700 nm emission, which can selectively adsorb the biological toxins such as ochratoxin and aflatoxin *in vivo* and *in vitro* (Liu J. et al., 2019). In this material, MIP has specific recognition ability for three biotoxins (sterigmatocystin, ochratoxin, and aflatoxin), and PLNPs act as fluorescent probes to eliminate background interference. In mice, the PLNPs@MIP in the tissues that contained biotoxins will have a brighter luminescence and a slower rate of being cleared out. In addition, the characteristic of persistent luminescence enabled PLNPs@MIP to realize the tracking of biological toxins, so as to explain the damage mechanism of biological toxins to human body. This work showed had a good application prospect in the detection of biological toxins in food.

## Bioimaging Based on Lanthanides-Doped PLPs

**Figure 2:**
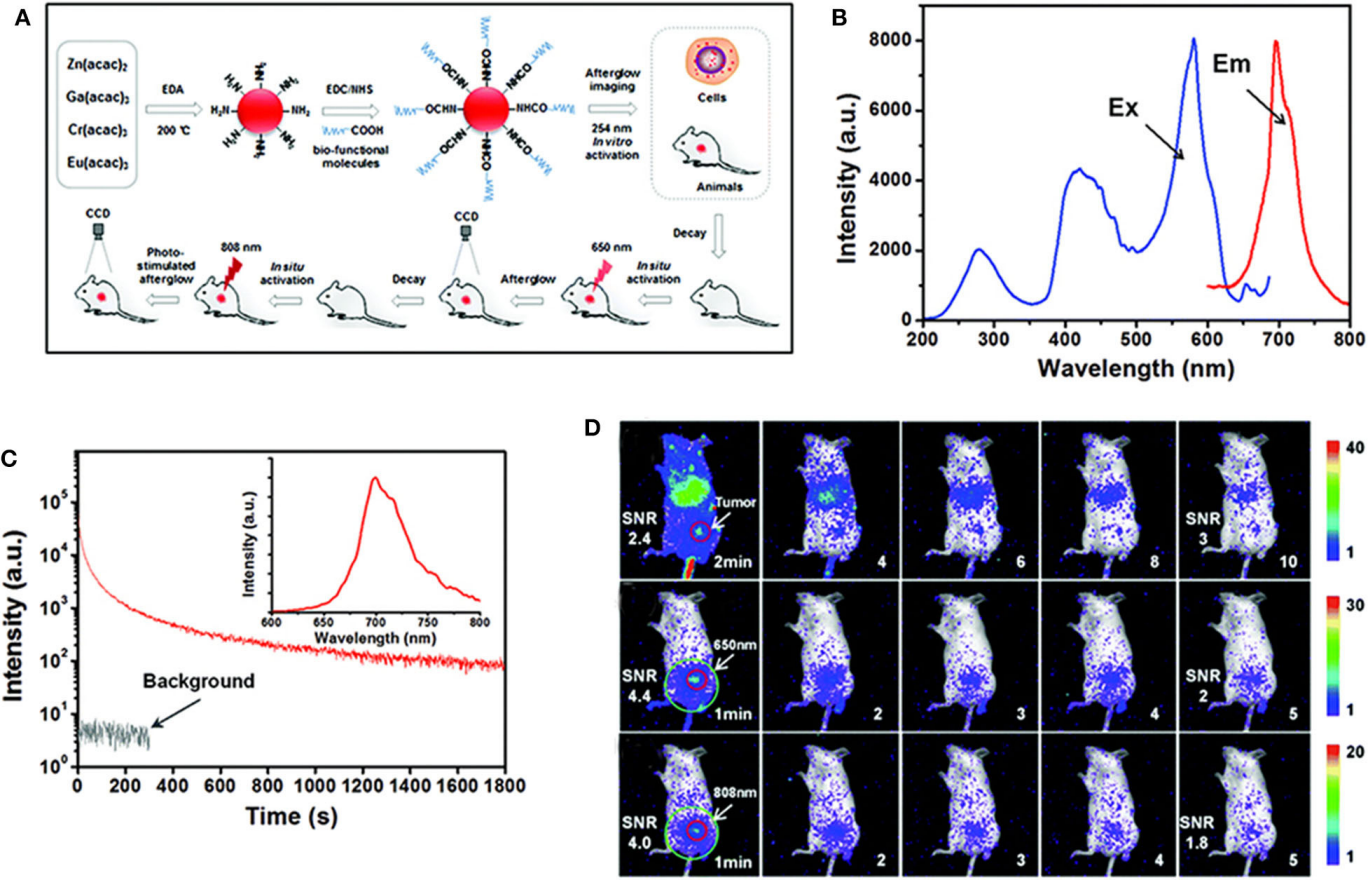
**(A)** Schematic diagram of synthesis and bioimaging application of the PLPs, **(B)** emission and excitation spectra of the PLPs, **(C)** decay curve of the PLPs of excitation at 254 nm and the inset is PersL spectrum, **(D)** bioimaging in mice after injection of PLPs. Reproduced from a literature Shi et al. (2016) with permission from Royal Society of Chemistry.

As mentioned in the first part, PLPs are ideal materials for bioimaging since they can effectively avoid spontaneous fluorescence of biological tissues (Abdukayum et al., 2013; Li et al., 2020). However, the PLPs synthesized by traditional high-temperature reactions lack appropriate modifiable group on the surface (Du et al., 2017). Moreover, the persistent luminescence of PLPs will diminish over time *in vivo*, and they cannot be effectively reactivated by UV light due to the limited penetration of UV. Shi et al. proposed a synthesis method for the preparation of amino functionalized ZnGa_2_O_4_:Cr,Eu PLPs with emission at 700 nm (Figures 2A–C) (Shi et al., 2016). The particle size and persistent luminescence of the PLPs can be controlled by changing the reaction conditions including time, pH, and so on. Due to the surface amino groups, the PLPs can be easily modified with biological molecules, such as folic acid for bioimaging. Lanthanides-doping in the PLPs significantly enhanced the persistent emission of Cr^3+^ at 700 nm. As a result, a high SNR (>4.0) was achieved in tumor imaging (Figure 2D). The authors also demonstrated that the PLPs can be re-excited *in vivo* to restore the signal intensity by NIR stimulation at 808 nm. This work paves the way for the development of PLPs with easy surface modification and *in-vivo* reactivation, showing a great application prospect in sustainable biological imaging.

Recently, Li et al. proposed a hydrothermal method for the synthesis of monodisperse Zn_1.25_Ga_1.5_Ge_0.25_O_4_:Cr^3+^,Yb^3+^,Er^3+^ PLPs with a broad emission from 650 to 850 nm (Li and Yan, 2016). These triple-doped PLPs had high quantum yields (9.86%) and long persistent luminescence time (>20 days). They found that after modifying folic acid on the surface, the particle that was low in biotoxicity can achieve targeted imaging of the tumor. Moreover, the therapy effect of oral administration was better than intravenous administration. The oral administration imaging based on this lanthanides-doped PLPs can improve the imaging time window while avoiding the injection step. These PLPs were expected to achieve effective optical imaging of oral drugs.

Terminal cancer is often difficult to treat because of the metastasis of cancer cells, so it is necessary to develop an imaging method to track the cancer metastasis in order to achieve more accurate treatment (Sengar et al., 2019). Lanthanides-doped PLPs have good promise in tumor tracking and metastasis imaging due to their bright and rechargeable persistent luminescence. Zhao et al. synthesized alginate containing PLPs Zn_1.1_Ga_1.8_G_e0.1_O_4_:Eu_0.009_,Cr_0.09_ with the emission at around 698 nm, and they covalently modified the PLPs with 4-carboxyphenylboric acid to target breast cancer cells (Zhao et al., 2020). The PLP probe was not interfered by spontaneous fluorescence and could achieve long-term tumor imaging. They found that the composite PLPs could target breast cancer cells precisely in mice by endocytosis. In addition, with the increase of labeled cells, the SNR increased as well. When the number of labeled cells was 10, the SNR is up to 3.0 ± 0.1. They also showed that these PLPs can be covalently modified with other targeted agents to achieve long-term tracking of other cancer metastasis. This tailored composite PLPs can solve the problems of cancer metastasis monitoring and provide a common platform for the accurate detection of cancer.

In bioimaging, the penetration depth of excitation light directly affects the imaging sensitivity in deep tissues (Rosticher et al., 2015; Zhong et al., 2019). Although X-rays have high energy and deep penetration, they are harmful to the human body (Song et al., 2017). So researchers hope to develop PLP probes that can be directly excited by NIR light. Xue et al. synthesized a kind of UC PLP (Zn_3_Ga_2_GeO_8_:Yb/Er/Cr). The PLPs have a NIR persistent luminescence emission at around 700 nm under NIR excitation and a duration of up to 15 h (Xue et al., 2017). Different from previous X-rays and UV excitation, NIR excitation shows less toxic effect on biological samples. The authors found can be recharged by stimulating light to recover the persistent luminescence *in vivo* for long-term bioimaging. This dual-mode NIR charging/emission imaging PLPs greatly improves the sensitivity and penetration depth of bioimaging, and the PLPs are expected to achieve long-term imaging of deep tissues.

## Drug Delivery Based on Lanthanides-Doped PLPs

In addition to bioimaging, PLPs can also be used as drug carriers to construct a bioimaging-guided drug delivery system (Zhang D. et al., 2018). Previous studies have shown that folate-modified PLPs can precisely target tumor cells, so the researchers attempted to load the PLPs with anti-tumor drugs to treat tumors *in situ* (Jabalera et al., 2020). Shi et al. synthesized the PLP probe Zn_1.1_Ga_1.8_Ge_0.1_O_4_:Cr^3+^,Eu^3+^@SiO_2_ with a 696 nm emission (Shi et al., 2015). The PLPs were covalently modified with folic acid and further loaded with anticancer drug doxorubicin to achieve targeted drug delivery. The PLP probe had strong NIR luminescence and long persistent time (>10 days). The authors showed that the probe was sensitive enough to tumor cells and could deliver drugs *in situ*. In addition, the PLPs can be readily recharged by excitation light, which can be applied in monitoring tumor cells for a long time for exploring the therapeutic mechanisms of antitumor drugs. This targeted drug delivery system has a good promise in tumor detection and cancer therapy.

Most PLP-based drug carriers have poor biocompatibility, so they are easy to be swallowed by macrophages in delivering drugs. In order to solve this problem, Liu et al. combined Zn_1.25_Ga_1.5_Ge_0.25_O_4_:Cr^3+^,Er^3+^,Yb^3+^ PLPs with membrane structure to prepare a new type of nanocarrier with biological characteristics (Liu et al., 2018). This nanocarrier has the NIR emission at around 700 nm, and displayed long persistent luminescence for over 20 days. In a proof of concept study, the erythrocyte membrane-coated nanocarriers effectively evaded the body's immune system and delivered drugs efficiently. The erythrocyte membrane-coated nanocarriers also retained the excellent luminescence performance of PLPs. Additionally, the authors found that the nanocarriers not only have good biocompatibility and bright persistent luminescence but also can achieve the release of drugs guided by bioimaging. This new drug carrier has a great prospect in targeted tumor therapy and other biological fields.

Addition to the poor biocompatibility, the drug-loading amount of PLPs is usually limited, which will affect the efficiency of drugs delivery and cancer therapy. Recently, Li et al. synthesized porous PLPs GdAlO_3_:Cr^3+^,Sm^3+^ for drug delivery (Li J. et al., 2018). These PLPs showed strong persistent luminescence at around 732 nm under UV excitation. They modified the surface of the PLPs with carboxymethyl chitosan to reduce their biotoxicity. The drug carriers based on this porous PLPs had high drug-loading efficiency. They also demonstrated in this composite that PLPs can achieve slow drug release when loaded with ibuprofen, an anti-inflammatory drug. These porous PLPs have a broad application prospect in drug delivery guided by bioimaging and can serve as a potential platform to explore the kinetics of drug release *in vivo*.

## Phototherapy Based on Lanthanides-Doped PLPs

Phototherapy has great advantages in tumor therapy because it has little toxicity to normal cells and can effectively kill cancer cells (Yang et al., 2018; Hu et al., 2019). The traditional photodynamic therapy (PDT) platform is usually composed of a fluorescent probe and photosensitizers. Due to its delayed luminescence, PLPs do not require long-term excitation light irradiation. Therefore, PLP-based fluorescent probes can effectively reduce the light damage to biological tissues and have a good application prospect in PDT. Most porphyrin-based photosensitizers are excited by UV light, but the long irradiation of UV is damage to biological tissues. To reduce the time of UV irradiation, Wang et al. synthesized Zn_1.25_Ga_1.5_Ge_0.25_O_4_:Cr^3+^,Yb^3+^,Er^3+^ PLPs for PDT (Wang et al., 2017). These PLPs had strong persistent luminescence at about 690 nm. They found that the persistent luminescence of these PLNPs can effectively activated the photosensitizers aluminum phthalocyanine to generate ^1^O_2_. They coated PLNPs with mesoporous silica to reduce its biotoxicity and conjugated photosensitizer to this the PLNPs to build the platform for PDT (The PLNPs were coated with mesoporous silica to reduce their biotoxicity and were further modified with the photosensitizer to construct the PDT platform). The authors demonstrated that the PDT platform can effectively eliminate cancer cells under short periods of UV irradiation. Therefore, the PLPs show good promise in UV-based PDT as the second excitation source to a photosensitizer and can provide possibilities for low-dose UV-excited PDT.

PLPs are often applied in phototherapy as fluorescent probes, but some PLPs do not have good water solubility, which affects their application in phototherapy. Homayoni et al. prepared Sr_2_MgSi_2_O_7_:Eu^2+^,Dy^3+^ PLPs with good water solubility by the sol-gel method, and used APTES to modify their surface (Homayoni et al., 2016). These PLPs had emission at around 660 nm under the X-ray excitation due to the ^4^F_9/2_-^6^H_13/2_ transitions of Dy^3+^. The PLPs were covalently modified with folic acid and were further modified with protoporphyrin, a photosensitizer, to achieve targeted PDT. The protoporphyrin was excited by the emission of PLPs in this PDT system, which enhanced the luminescence of protoporphyrin by 10 times. By integrating *in-situ* biological imaging and photodynamic therapy, this PDT system was able to produce ^1^O_2_ continuously upon the primary X-ray excitation to achieve efficient tumor therapy. This work showed the good promise of these PLPs in tumor phototherapy and radiotherapy.

Besides PDT, photothermal therapy (PTT) is also an effective phototherapy method, and the traditional PTT platform is usually composed of fluorescent probe and photothermal agent (Zheng et al., 2016; Zhao et al., 2017; Zhen et al., 2018). Different from the previous method of carrying photothermal agent, Wu et al. synthesized a Zn_3_Ga_2_SnO_8_:Cr^3+^,Nd^3+^,Gd^3+^ PLPs with photothermal effect (Wu et al., 2016). These PLPs had strong persistent luminescence at about 700 nm because of the ^2^E–^4^A_2_ transition of Cr^3+^. This use of PLPs that don't need the prolonged exposure of high-energy excitation light could effectively reduce light damage in PTT. The doped Nd^3+^ can absorb the energy of excitation light at 808 nm and convert it into heat energy for ablating the tumor cells. Due to the combination of persistent luminescence and photothermal effect, this PLP-based integrated platform is expected to realize efficient PTT guided by bioimaging, which can effectively reduce the side effects of PTT. This work provides a reference for the designing of non-composite PTT platform.

## Conclusion

Lanthanides ions are often doped as luminescence centers or defects to regulate the persistent luminescence of PLPs. With proper lanthanides doping, the researchers have synthesized many kinds of PLPs with NIR persistent luminescence. These PLPs can effectively avoid spontaneous luminescence in tissues, and NIR luminescence allows PLPs to have deeper penetration. Therefore, the lanthanides-doped PLPs are ideal materials for biosensing, bioimaging, and cancer therapy. Various synthetic methods and modification strategies were proposed to improve the water solubility and biocompatibility of the PLPs. The PLP-based composite platforms have a broader prospect in biomedicine applications. In this paper, the synthesis and biomedical applications, including biosensing, bioimaging, drug delivery, and phototherapy of lanthanides-doped PLPs with NIR emission, are reviewed, aiming to provide instructions for the further studies on lanthanides-doped PLPs.

Although PLPs with NIR emission have great promise in biomedicine, great challenges are still confronted by PLPs before their practical applications. Currently, PLPs with NIR emission are generally synthesized with “top-down” methods, such as wet grinding, particularly for PLPs with emission in the NI-II region. Such PLPs usually show irregular size/shape, poor dispersibility, and surface modification. The “bottom-up” methods need to be developed for the controlled synthesis of PLPs with NIR emission. On the other hand, the PLPs with NIR emission are usually activated by UV light. The UV light has shallow tissue penetration and usually causes serious photo damage to tissues. Developing PLPs that can be directly charged by NIR light is highly desired for deep-tissue imaging and therapy. Last but not the least, research about the biosafety of the PLPs with NIR emission is rather limited. Many efforts have to be made to systematically investigate the biosafety of PLPs. With further research, these challenges are expected to be addressed, and the lanthanides-doped PLPs with NIR emission will be readily implemented into the clinical workflow for disease diagnosis and therapy.

## Author Contributions

QY supervised the project and mainly wrote the paper. XQ and JW co-wrote the paper. All authors discussed the reviewed results and commented on the manuscript.

## Conflict of Interest

The authors declare that the research was conducted in the absence of any commercial or financial relationships that could be construed as a potential conflict of interest.
